# A novel stop-gain pathogenic variant in the *KCNQ1* gene causing long QT syndrome 1

**DOI:** 10.1186/s40001-023-00984-0

**Published:** 2023-01-12

**Authors:** Samira Kalayinia, Mohammad Dalili, Maryam Pourirahim, Majid Maleki, Nejat Mahdieh

**Affiliations:** 1 Cardiogenetic Research Center, Rajaie Cardiovascular Medical and Research Center, Iran University of Medical Sciences, Tehran, Iran; 2 Rajaie Cardiovascular Medical and Research Center, Iran University of Medical Sciences, Tehran, Iran

**Keywords:** Long QT syndrome 1, Genetic, *KCNQ1*, Whole-exome sequencing, Variant

## Abstract

**Background:**

Inherited primary arrhythmias, such as long QT (LQT) syndromes, are electrical abnormalities of the heart mainly due to variants in 3 genes. We herein describe a novel stop-gain pathogenic variant in the *KCNQ1* gene in an Iranian child with LQT syndrome 1.

**Methods:**

The patient and his family underwent clinical evaluation, electrocardiographic Holter monitoring, and whole-exome sequencing. Sanger sequencing and segregation analysis were used to confirm the variant in the patient and his family, respectively. The pathogenicity of the variant was checked via an in silico analysis.

**Results:**

The proband suffered from bradycardia and had experienced syncope without stress. The corrected QT interval was 470 ms (the Schwartz score ≥ 3.5), and the Holter monitoring showed sinus rhythm, infrequent premature atrial contractions, and a prolonged QT interval in some leads. Whole-exome and Sanger sequencing showed c.968G > A in 3 affected family members. According to the American College of Medical Genetics and Genomics criteria, c.968G > A was classified as a pathogenic variant.

**Conclusions:**

The *KCNQ1* gene is the main cause of LQT syndromes in our population. The common genes of LQT syndromes should be studied in our country’s different ethnicities to determine the exact role of these genes in these subpopulations.

## Introduction

Long QT (LQT) syndromes are inherited primary arrhythmias affecting 1 in every 5000 to 20000 neonates worldwide [13]. They are characterized by the prolongation of the corrected QT interval on the electrocardiogram (ECG), and they propagate the risk of ventricular arrhythmias, resulting in torsade de pointes [16]. LQT syndromes occur due to alterations in the expression and/or function of repolarizing ionic channels. The main clinical features of LQT syndromes usually include palpitations, syncope, seizures, and ventricular arrhythmias, increasing the risk of sudden cardiac death [12].

The congenital form of LQT syndromes is mostly caused by autosomal dominant pathogenic variants in K + channel proteins, namely Kv7.1 and Kv11.1, in cardiac cells. Kv7.1 and Kv11.1 channels play a significant role in delayed-rectifier K + currents, required for normal ventricular repolarization. Kv7.1 is encoded by the *KCNQ1* gene (LQT syndromes type 1 or LQT1) [1]. Pathogenic variants in *KCNQ1* are the most common cause of congenital LQT syndromes. Without medical treatment, the mortality rate is high within 1 year after the first syncope episode, [11] but the rate decreases significantly during a 15-year follow-up [13]. LQT syndromes can be diagnosed by clinical features, QT-interval prolongation on 12‐lead ECGs, provocative tests such as epinephrine infusion and exercise stress, family history, and genetic testing.

We have previously described the genetic spectrum of the common types of LQT syndromes in our population. [5, 8, 17]. Here, we report a novel stop-gain pathogenic variant in *KCNQ1* causing LQT1 in an Iranian child.

## Methods

### Clinical evaluation of family recruitment and ethics approval

**Fig. 1:**
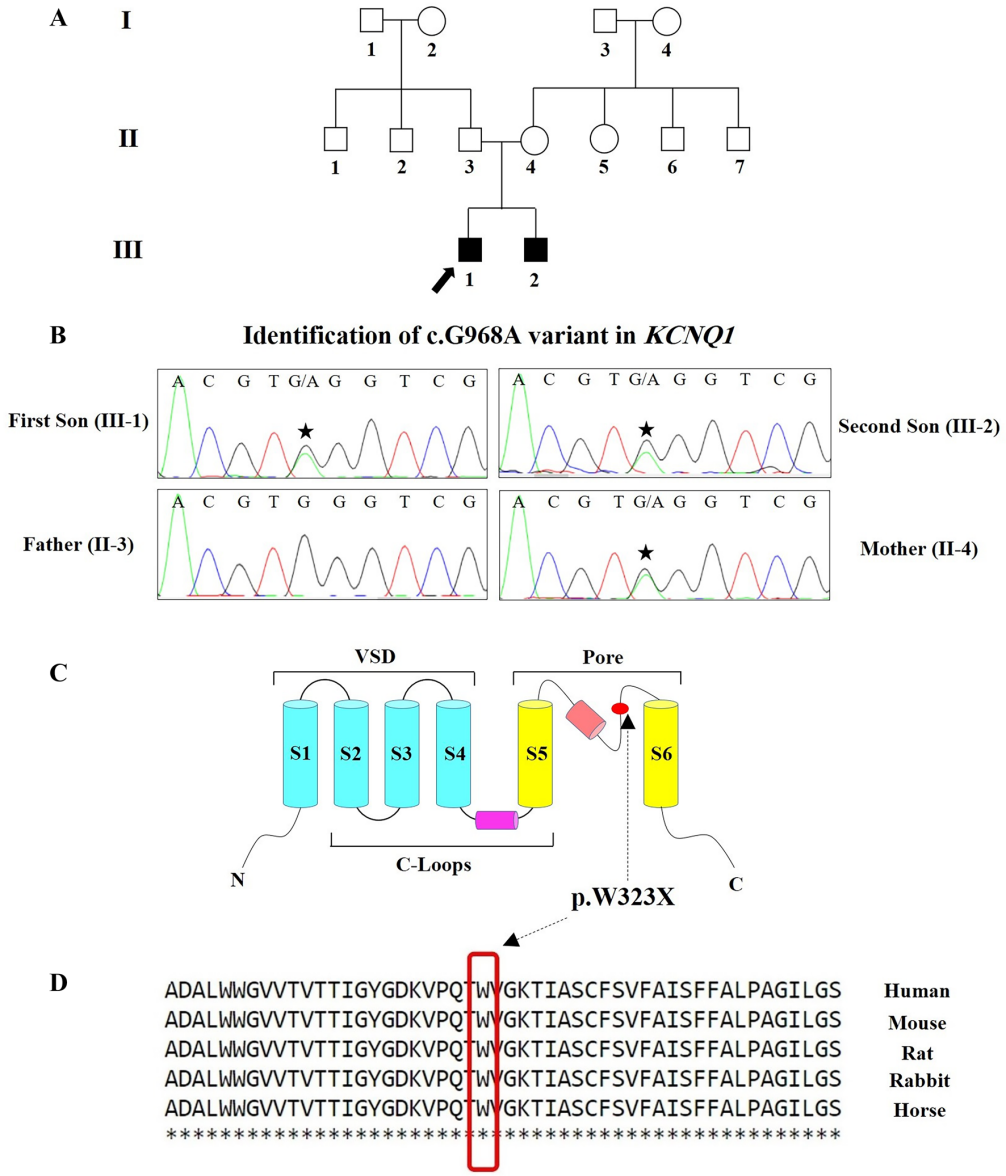
The image presents the pedigree of the family with long QT (LQT) syndromes, as well as the results of the electrocardiogram (ECG) and sequencing chromatograms of the mutated nucleotide in the *KCNQ1* gene. **A** The pedigree of the family with LQT syndromes is presented herein. The proband is indicated with the arrow. **B** The Sanger sequencing results of the *KCNQ1* gene in the patient and his family members are shown here. The patients carried a heterozygous nonsense variant: c.G968A. **C** The image demonstrates the poor region of the Kv11.1 schematic structure with the W323X variant. **D** This region includes amino acids conserved among humans, mice, rats, rabbits, and horses

An Iranian family recruited in this study, had been referred to Rajaie Cardiovascular Medical and Research Center, Tehran, Iran, for the experience of recurrent syncope of its children. The proband was a 10-year-old boy with a 3-year-old brother (Fig. 1A-, III-1 & III-2, respectively). The parents were unrelated and healthy: the father was 41 years old, and the mother was 34. Routine cardiovascular examinations, including echocardiography, 12-lead ECGs, exercise tests, and 24-h Holter monitoring, were carried out for all the family members, affected or healthy. The patients (Fig. 1A, III-1 & III-2) underwent an electrophysiological examination with a 2-electrode voltage−clamp amplifier (TEC10CD, NPI Electronics, Tamm, Germany) featuring KCl-filled electrodes of about 0.8 MU resistance [18]. The currents were measured at room temperature by applying bath solutions, including 10 mM of HEPES (pH 7.2), 105 mM of NaCl, 1.8 mM of CaCl2, and 10 mM of KCl. The Bazett formula was used for calculating the heart rate QT intervals. The study was performed due to the Declaration of Helsinki and was approved by the Ethics Committee of Rajaie Cardiovascular Medical and Research Center (IR.RHC.REC.1401.054). Informed consent was achieved from all participants and from the parents of participants below 16-year-old.

### Whole-Exome sequencing and in Silico analysis

Blood samples of family members were obtained. Genomic DNA extraction was performed using the DNSol Midi Kit (Roche: Product No. 50072012). Whole-exome sequencing was done on the proband (Fig. 1A, III-I) at Macrogen (Amsterdam, the Netherlands). Enrichment and capture of all exones were carried out using SureSelect Exon V7 Library Prep Kit. Exome was sequenced on an Illumina HiSeq 6000 machine based on the manufacturer’s protocol. A read quality value of greater than 20 and a depth of greater than 7 was used for the next steps. The quality of the reads was surveyed with FastQC. The clean reads were aligned to the reference genome (UCSC Genome Browser, hg19) applying the Burrows–Wheeler Aligner (BWA-MEM v.07.17) [3]. Insertion and/or deletion and single-nucleotide polymorphism were called with the aid of the Genome Analysis Toolkit (GATK, v.4.1.4.1) [6]. Annotation of determined variants was done by ANNOVAR [15] and filtered according to the Exome Aggregation Consortium (ExAC), the 1000 Genomes Project, Exome-Sequencing Project ESP6500, and the Genome Aggregation Database (gnomAD) minor allele frequency (MAF) of 0.001. The candidate variants were investigated with bioinformatics tools, consisting of MutationTaster (www.mutationtaster.org), CADD (cadd.gs.washington.edu), PROVEAN (provean.jcvi.org), SIFT (https://sift.bii.a-star.edu.sg), and PolyPhen-2 (genetics.bwh.harvard.edu/pph2) according to the 2015 guidelines of the American College of Medical Genetics and Genomics (ACMG) [10]. Moreover, the conservation of the variants regions were analyzed using the GERP +  + score and CLUSTALW Web Server (https://www.genome.jp/tools-bin/clustalw) by comparing the amino acid sequences of different species.

### Variant validation and segregation analysis

The identified candidate variant, *KCNQ1* c.968G > A: p.Trp323, was confirmed and segregated using polymerase chain reaction (PCR) and Sanger sequencing to assess the family members (healthy/patient).

Primer pair was designed using the Primer3 v.04.0 (http://bioinfo.ut.ee/primer3-0.4.0/) with the sequences: forward: 5ʹ-TGCTCTTTGTTGACGACCA-3′ and reverse: 5′-AGCGTGGAAGTGCCCTCT-3′. PCR was carried out on a SimpliAmp Thermal Cycler (Thermo Fisher Scientific) with 1.5 mmol/L of MgCl2, 10 pmol/L of the primers, 200 mmol/L of dNTP, 100 ng DNA, and 1 U of Taq DNA polymerase (Amplicon, UK). Thereafter, incubation at 95 °C for 5 min and 35 amplification cycles (30 s at 95 °C, 30 s at 62 °C, and 30 s at 72 °C) was applied. The products of the PCR were sequenced on an ABI Sequencer 3500XL PE (Applied Biosystems), and the sequences were surveyed with CodonCode Aligner (v.7.1.2) (Fig. 1B).

## Results

### Clinical findings

**Fig. 2:**
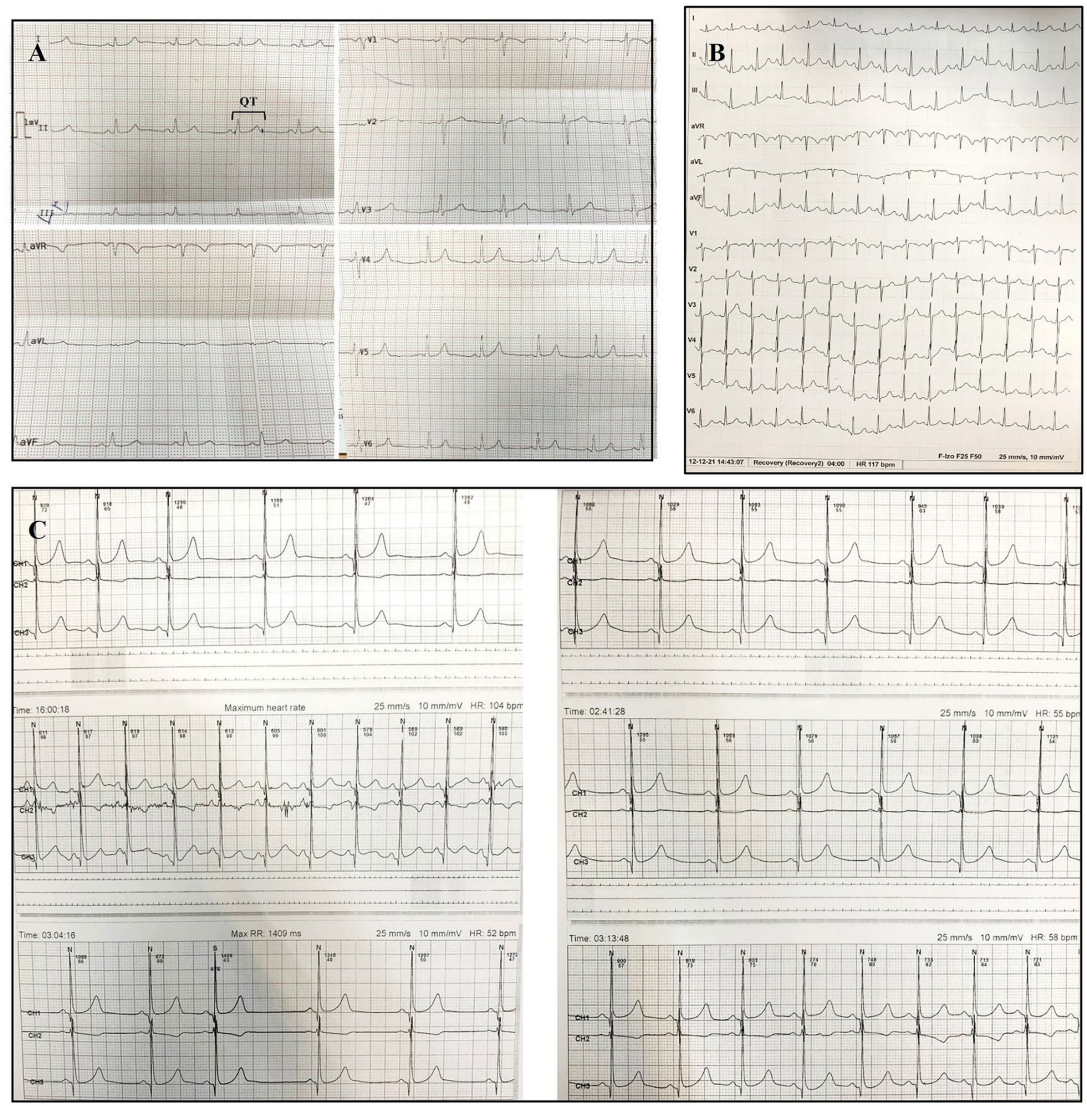
**A** The baseline 12-lead standard electrocardiogram indicates a normal sinus rhythm, a normal QRS frontal axis, and a normal corrected QT interval. **B** Four minutes after exercise cessation, the corrected QT interval is about 480 ms, deemed prolonged for this situation. **C** The image presents the leads of ambulatory monitoring. The corrected QT interval is normal in most of the leads but is prolonged in the right lower panel

The proband (Fig. 1A, III-1) had suffered 2 syncope episodes without stress in the past year. Sinus rhythm, infrequent premature atrial contractions, and a prolonged QT interval in some leads were detected in his Holter recording (Fig. 2A, C). The minimum Schwartz score [2] was 3.5, indicating the high probability of LQT syndromes.

Concerning the family history, the younger brother (Fig. 1A, III-2) also had suffered syncope and exhibited similar symptoms. The mother (Fig. 1A, II-4) had experienced chest pain and dyspnea in the preceding 5 months (no notable ECG manifestations). The father (Fig. 1A, II-3) had no history of arrhythmia or syncope, with no signs of heart disease or aberrant ECGs in his clinical examinations.

### Molecular findings

Whole-exome sequencing was performed on the proband (Fig. 1A, III-1) to discover the causative variant. A novel stop-gain pathogenic variant, c.968G > A, was found in the seventh exon of *KCNQ1*. This variant substituted tryptophan at site 323 for a stop codon, proposed to cause a premature KCNQ1 truncated protein and/or nonsense-mediated KCNQ1 mRNA decay. Notably, the variant has not yet been reported either in the 1000 Genomes Project, ExAc, gnomAD, HGMD, and ClinVar or in publications. According to the ACMG, c.968G > A was determined as a pathogenic variant (criteria: PVS1, PM2, PP1, and PP4). This nonsense variant was considered the cause of the disease by SIFT, PolyPhen-2, PROVEAN, FATHMM, and GERP +  + . The CADD Phred score was 41. The variant was confirmed in the proband (Fig. 1A, III-2) by PCR and Sanger sequencing in the heterozygous state. It was also detected in the proband’s affected brother (Fig. 1A, III-2) and the suspected mother (Fig. 1A, II-4) as a heterozygote. The father (Fig. 1A, II-3) had a normal sequence at this position (Fig. 1B). DNA from the other pedigree members was unavailable. A schematic secondary structure of the KCNQ1 protein is presented in Fig. 1C. In addition, based on the CLUSTALW results, tryptophan323 was located in the conserved part of the KCNQ1 protein (Fig. 1D).

## Discussion

In many populations, inherited LQT syndromes (~ 75% of LQT syndromes) occur due to variants of 3 genes: *KCNQ1* (~ 35%), *KCNH2* (~ 30%), and *SCN5A* (~ 10%), which encode Kv7.1, Kv11.1, and Nav1.5, respectively [14]. However, other major genes might exist in some populations. For instance, in our previous study, we demonstrated that the common genes of LQT syndromes were responsible for only 43% of patients in a sample of the Iranian population [5]. Here, we describe an Iranian child suffering from syncope without stress and with a minimum Schwartz score of 3.5 due to a novel nonsense variant in *KCNQ1*.

Nonsense variants account for a lower percentage of point mutations in *KCNQ1*, with approximately 10% of *KCNQ1* variants being nonsense [5]. In the present study, we found a novel nonsense variant, c.968G > A, leading to p.Trp323Ter in *KCNQ1* in an Iranian proband whose brother and mother were also symptomatic. Notably, p.Trp323 is a hotspot in this gene because p.Trp323Ter due to c.969G > A (not c.968G > A) was reported in a patient by [4]. Our patients had bradycardia, a prolonged corrected QT interval (470 ms), sinus rhythm, infrequent premature atrial contractions, and a prolonged QT interval in some leads.

The pathogenic variant introduced herein may lead to more severe phenotypes, although a genotype–phenotype correlation with a significant number of affected individuals is required to elucidate the effects of this variant on clinical presentations and arrhythmic events. Our in silico analyses showed that this variant is pathogenic, and p.Trp323 is a conserved position among different species. Further, p.Trp323 is located in a region of poor channel condition, so any change in this position could have a potential pathogenic effect on the function of the channel such that the I_Ks_ (K slowed delayed rectifier) current is severely affected. As shown, any dysfunction in this current may lead to a prolonged cardiac action potential and U-wave and T-wave changes and trigger torsade de pointes [9].

Chain termination variants, such as nonsense variants, lead to truncated proteins and severe symptoms, whereas missense variants result in amino acid substitutions. Depending on their location and effects on messenger RNAs and proteins, splicing variants may have variable phenotypes in patients with such variants. We recommend further research on the effects of different *KCNQ1* variants on symptoms and signs among patients with chain termination, missense, and splicing variants. In this regard, we have previously reported the effects of the splicing variant in the *MYO15A* gene [7].

Our findings suggest that *KCNQ1* might be the principal cause of LQT in our population. There are many ethnicities in our country; the common genes of LQT syndromes should, therefore, be studied in these different ethnicities to determine the exact role of these genes in these subpopulations.

## Data Availability

The datasets generated and/or analyzed during the current study are available in the ClinVar repository [https://www.ncbi.nlm.nih.gov/clinvar/variation/1723453/?new_evidence=false]. The accession number of the variant in ClinVar is as follows: NM_000218.3 (KCNQ1):c.968G > A (p.Trp323Ter): VCV001723453.1.
